# Assessment of Selected Baseline and Post-PCI Electrocardiographic Parameters as Predictors of Left Ventricular Systolic Dysfunction after a First ST-Segment Elevation Myocardial Infarction

**DOI:** 10.3390/jcm10225445

**Published:** 2021-11-22

**Authors:** Tomasz Fabiszak, Michał Kasprzak, Marek Koziński, Jacek Kubica

**Affiliations:** 1 Department of Cardiology and Internal Medicine, Collegium Medicum, Nicolaus Copernicus University, ul. Skłodowskiej-Curie 9, 85-094 Bydgoszcz, Poland; medkas@o2.pl (M.K.); jwkubica@gmail.com (J.K.); 2 Department of Cardiology and Internal Medicine, Medical University of Gdańsk, ul. Powstania Styczniowego 9B, 81-519 Gdynia, Poland; marek.kozinski@gumed.edu.pl

**Keywords:** myocardial infarction, ECG, risk stratification, left ventricular systolic dysfunction, primary PCI

## Abstract

Objective: To assess the performance of ten electrocardiographic (ECG) parameters regarding the prediction of left ventricular systolic dysfunction (LVSD) after a first ST-segment-elevation myocardial infarction (STEMI). Methods: We analyzed 249 patients (74.7% males) treated with primary percutaneous coronary intervention (PCI) included into a single-center cohort study. We sought associations between baseline and post-PCI ECG parameters and the presence of LVSD (defined as left ventricular ejection fraction [LVEF] ≤ 40% on echocardiography) 6 months after STEMI. Results: Patients presenting with LVSD (*n* = 52) had significantly higher values of heart rate, number of leads with ST-segment elevation and pathological Q-waves, as well as total and maximal ST-segment elevation at baseline and directly after PCI compared with patients without LVSD. They also showed a significantly higher prevalence of anterior STEMI and considerably wider QRS complex after PCI, while QRS duration measurement at baseline showed no significant difference. Additionally, patients presenting with LVSD after 6 months showed markedly more severe ischemia on admission, as assessed with the Sclarovsky-Birnbaum ischemia score, smaller reciprocal ST-segment depression at baseline and less profound ST-segment resolution post PCI. In multivariate regression analysis adjusted for demographic, clinical, biochemical and angiographic variables, anterior location of STEMI (OR 17.78; 95% CI 6.45–48.96; *p* < 0.001), post-PCI QRS duration (OR 1.56; 95% CI 1.22–2.00; *p* < 0.001) expressed per increments of 10 ms and impaired post-PCI flow in the infarct-related artery (IRA; TIMI 3 vs. <3; OR 0.14; 95% CI 0.04–0.46; *p* = 0.001) were identified as independent predictors of LVSD (Nagelkerke’s pseudo R^2^ for the logistic regression model = 0.462). Similarly, in multiple regression analysis, anterior location of STEMI, wider post-PCI QRS, higher baseline number of pathological Q-waves and a higher baseline Sclarovsky-Birnbaum ischemia score, together with impaired post-PCI flow in the IRA, higher values of body mass index and glucose concentration on admission were independently associated with lower values of LVEF at 6 months (corrected R^2^ = 0.448; *p* < 0.00001). Conclusions: According to our study, baseline and post-PCI ECG parameters are of modest value for the prediction of LVSD occurrence 6 months after a first STEMI.

## 1. Introduction

Electrocardiography (ECG), invented by Willem Einthoven nearly 120 years ago, remains one of the essential diagnostic modalities in cardiology [1], shaping the elementary division of acute coronary syndromes into those with and without persistent ST-segment depression, affecting the timing and mode of management and adding to short- and long-term risk stratification [2,3,4].

It is estimated that left ventricular systolic dysfunction (LVSD), recognized as a long-term consequence of myocardial infarction (MI), may affect up to 60% of post-MI patients [5]. Its occurrence mainly depends on the presence of frozen myocardium, size of post-MI necrosis, and occurrence of left ventricular remodeling [6,7].

Left ventricular ejection fraction (LVEF), measured with echocardiography, is by far the most popular method for diagnosing LVSD in the clinical setting [8].

LVSD is a well-recognized marker of unfavorable prognosis in post-MI patients [8], translating into a 3–4-fold increase in mortality and higher rates of cardiovascular adverse outcomes, such as cardiac rupture, sudden cardiac arrest, recurrent myocardial infarction, ventricular arrhythmias, stroke, prolonged hospitalization and rehospitalization [7,9,10,11]. The mortality rate among post-MI patients with asymptomatic LVSD after 12 months of MI is as high as 12% and amounts to 36% in symptomatic patients [12]. LVSD independently predicts short-, mid- and long-term mortality after MI [12,13,14,15].

There are many reports regarding the predictive value of ECG with respect to the development of LVSD after STEMI [4,16,17]. A vast part of these reports however, comes from the era of thrombolytic treatment of STEMI and was derived from non-uniform cohorts of patients regarding forms of MI, reperfusion treatment and pharmacotherapy. Nowadays, in consequence of current standards of STEMI management, incorporating percutaneous coronary intervention (PCI) as a means of effective and safe reperfusion, together with dual antiplatelet treatment, we have witnessed a spectacular reduction in the rates of death, reinfarction, heart failure and strokes.

Our investigation aims to assess the relationship between selected baseline and post-PCI ECG variables and the presence of LVSD 6 months after a first STEMI.

## 2. Methods

### 2.1. Study Design

The investigation was a prospective cohort trial including patients receiving primary PCI with stent implantation for a first STEMI. Study design, including the inclusion and exclusion criteria, was described in detail in our previous publication exploring associations of ECG with post-MI left ventricular remodeling (LVR) [18]. Here, we provide only a brief overview of the study design. Major exclusion criteria were as follows: any previous myocardial infarction or coronary revascularization, presence of advanced acute or chronic heart failure (defined as class IV according to the Killip classification or class ≥III according to the New York Heart Association), presence of ECG abnormalities that might become study confounders (i.e., left bundle branch block, isolated posterior myocardial infarction, isolated right ventricular myocardial infarction, permanent atrial fibrillation), severe valvular heart disease, any cardiomyopathy, poorly controlled arterial hypertension (defined as blood pressure ≥180/110 mmHg on hospital admission) and significant kidney dysfunction on hospital admission (defined as creatinine concentration exceeding 2 mg/dL).

The analyzed ECG parameters included:

1. heart rate,
2. location of STEMI,
3. number of leads with ST-segment elevation,
4. sum of ST-segment elevation in all leads,
5. maximal ST-segment elevation in a single lead,
6. ST-segment resolution,
7. presence of reciprocal ST-segment depression ≥0.1 mV on admission to hospital,
8. number of leads with pathological Q-waves [19],
9. Sclarovsky-Birnbaum ischemia score [2],
10. QRS complex duration.

The primary study endpoint was the occurrence of LVSD 6 months after STEMI. LVSD was defined as LVEF ≤40% on transthoracic echocardiography. This cut-off value was previously shown to be associated with unfavorable prognosis [10,20,21,22,23,24]. Additionally, LVEF ≤40% is used by the European Society of Cardiology guidelines for defining heart failure with reduced ejection fraction [25] and post-infarct patients who benefit from therapy with a beta-blocker, angiotensin-converting enzyme inhibitor or mineralocorticoid receptor antagonist [26].

First, we planned to compare the clinical, biochemical, angiographic and echocardiographic characteristics. We also assessed differences in ECG parameters between patients with and without LVSD 6 months after STEMI. Second, we prespecified uni- and multivariate analyses aimed at identifying predictors of post-infarct LVSD. A particular focus was placed on the investigated ECG parameters.

As a final step, we planned to check whether the variables predictive of the primary study endpoint were associated with lower values of LVEF (expressed as a continuous parameter) 6 months after STEMI.

Details of coronary angiography, PCI technique and ECG evaluation were also published in our previous publication [18]. Importantly, we aimed to restore normal blood flow in the infarct-related artery (IRA) during the primary PCI. Other non-culprit lesions of ≥90% in major coronary vessels were treated during the index hospitalization, while PCIs of the remaining significant stenoses (70–90%) were done electively (within 1 month of STEMI occurrence).

Informed consent for participation in the study was obtained from each participant. The study received approval from the local Bioethics Committee of Collegium Medicum, Nicolaus Copernicus University in Toruń (protocol code KB 440/2004). Throughout the entire course of the study, the Declaration of Helsinki and the principles of good clinical practice were applied.

### 2.2. Echocardiographic Assessment

Two-dimensional transthoracic echocardiography was performed in order to evaluate left ventricular systolic function using a Philips Sonos 7500 device (Philips, Andover, MA, USA) at two time points: before hospital discharge and after 6 months. Image acquisitions and measurements were performed according to the recommendations of the European Association of Echocardiography and the American Society of Echocardiography [27,28]. The biplane method of discs (modified Simpson’s rule) based on apical 4-chamber and 2-chamber view was utilized for LVEF estimation. The echocardiographer was blinded to the ECG analysis. The intra-observer coefficient of variation for LVEF estimation for the first 50 patients was 2.5%.

### 2.3. Data Collection and Statistical Analysis

Relevant data were collected and initially analyzed using Microsoft Excel spreadsheet software (Microsoft Corporation, Redmond, WA, USA). No missing data were present.

Descriptive analysis was used to summarize participant characteristics. Categorical data are presented as frequencies and percentages. Continuous variables are reported as medians and interquartile ranges. Correspondence with normal distribution was verified with the Shapiro-Wilk test. Between-group differences were tested using the Mann-Whitney U test for continuous variables and the Pearson Chi-square and Mantel-Haensztel tests for categorical variables. In order to identify predictors of LVSD at 6 months, logistic regression was used. The results are presented as odds ratios (OR) with 95% confidence intervals. Only variables with univariate *p*-values of <0.1 were included in the multivariate models. Stepwise backward selection was employed to select variables included in the best-fitting models. To identify predictors of LVEF at 6 months, we used multiple linear regression. Variables showing univariate *p*-values of <0.1 were considered eligible for multivariate analyses. The variables were then removed via stepwise backward selection. *p*-values of <0.05 were considered significant. Data analysis was conducted using Statistica version 13 (TIBCO Software Inc., Palo Alto, CA, USA) and SPSS version 23 (IBM, Armonk, NY, USA).

## 3. Results

### 3.1. The Course of the Study

The final analysis included 249 patients. A detailed description of the course of the study can be found in our previous publication [18].

### 3.2. Clinical, Demographic, Angiographic and Biochemical Parameters

The study cohort was primarily composed of middle-aged men. At baseline, patients who presented with LVSD after 6 months of follow-up showed a higher prevalence of diabetes mellitus, left anterior descending artery (LAD) as the IRA and TIMI 0 flow before PCI, but less frequent TIMI 3 flow post PCI. Slightly worse kidney function (assessed based on glomerular filtration rate), higher plasma glucose concentration on admission to hospital, larger enzymatic infarct size (as assessed with maximal concentration of troponin I and maximal activity of isoenzyme MB of creatinine kinase [CK-MB]), higher concentration of B-type natriuretic peptide (BNP) and more common usage of GPIIb/IIIa inhibitors during PCI could also be found in this group. Detailed characteristics of the study population are presented in Table 1.

### 3.3. Echocardiographic Characteristics

Table 2 presents major echocardiographic parameters at the time of discharge from hospital and after 6 months in the overall study population and in the subgroups with and without LVSD. Within 6 months of STEMI, a significant increase in median values of LVEF from 44% to 46% could be noted, leading to a decline in the percentage of patients with LVEF ≤40% from a baseline value of 33.7% to 20.9% after 6 months (*p* < 0.001; Table 3).

Interestingly, patients with LVEF ≤ 40% at the time of discharge from hospital, but not 6 months after STEMI (*n* = 39), when compared with those presenting with LVEF ≤40% both at hospital discharge and LVSD 6 months after STEMI (*n* = 45), had a lower proportion of the LAD as the IRA (31 [79.5%] vs. 42 [93.3%]; *p* = 0.058), more frequent TIMI 3 flow in the IRA following PCI (38 [97.4%] vs. 34 [75.6%]; *p* = 0.002) and lower values of cardiac biomarkers, including maximal concentration of cardiac troponin I (50.0 [27.7–50.0] vs. 50.0 [50.0–50.0] ng/mL; *p* = 0.039), maximal activity of CK-MB (354 [159–404] vs. 555 [378–761] U/L; *p* < 0.001) and BNP concentration on hospital discharge (177.3 [113.5–282.0] vs. 439.3 [233.0–751.5] pg/mL; *p* < 0.001).

### 3.4. Electrocardiographic Characteristics

Detailed baseline and post-PCI electrocardiographic data are reported in Table 4.

### 3.5. Characteristics Comparison of Patients with and without LVSD

As reported in Table 1, both groups showed no significant demographic nor clinical differences, except for a higher prevalence of diabetes in the LVSD (+) group. LVSD (+) patients also presented a less favorable angiographic profile, including more frequent involvement of LAD as the IRA, more widespread usage of GP IIb/IIIa inhibitor, a higher incidence of TIMI 0 and less frequent occurrence of TIMI 3 flow before and after PCI, respectively. Patients who presented with LVSD after 6 months were also characterized at baseline by worse renal function as assessed with glomerular filtration rate, higher blood glucose concentration on admission, more extensive release of myocardial necrosis markers and higher concentrations of B-type natriuretic peptide both on admission and at discharge. At discharge, both groups were receiving similar pharmacological treatment regarding aspirin, clopidogrel, statin, beta-blocker and ACEI/ARB (all used in ≥98.5% of patients); however, LVSD (+) patients were receiving aldosterone antagonist (28.8% vs. 5.1%; *p* < 0.001) and diuretic (28.8% vs. 4.6%; *p* < 0.001) more frequently than their LVSD (–) counterparts.

### 3.6. Electrocardiographic Characteristics of Patients with LVSD

**Figure 1:**
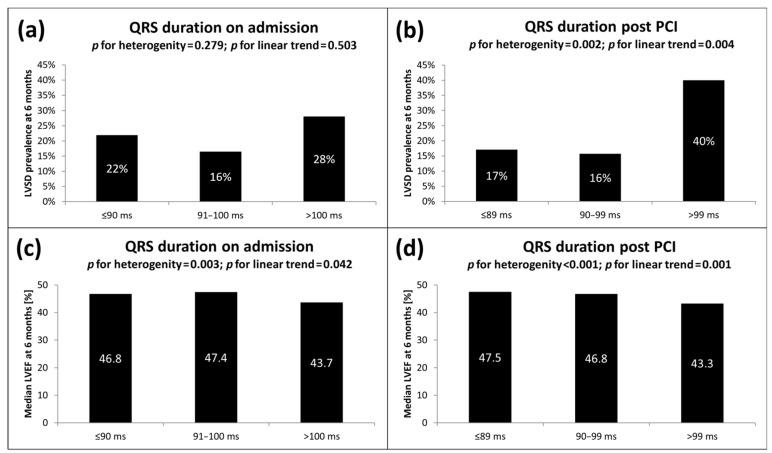
LVSD prevalence 6 months after STEMI according to terciles of QRS duration (**a**) on admission and (**b**) post PCI. Median LVEF 6 months after STEMI according to increasing terciles of QRS duration (**c**) on admission and (**d**) post PCI. LVEF, left ventricular ejection fraction; LVSD, left ventricular systolic dysfunction; ms, milliseconds; PCI, percutaneous coronary intervention; STEMI, ST-segment elevation myocardial infarction.

The analyzed ECG parameters, both at baseline and post PCI, point to more severe ischemia and a more extensive MI in the LVSD (+) group. These include faster heart rate, more widespread ST-segment elevation and Q-wave development and higher total and maximal ST-segment elevation. More pronounced ischemia in LVSD (+) patients was also evidenced by a higher incidence of anterior wall location, reciprocal ST-segment depression ≥1 mm and grade 3 according to Sclarovsky-Birnbaum ischemia grading system at baseline. In post-PCI ECG assessment, lower incidence and degree of ST-segment resolution and longer duration of the QRS complex were associated with the presence of LVSD after 6 months. A detailed comparison of ECG parameters is reported in Table 4. As shown in Figure 1, we also found visual variability and a linear trend toward an increasing rate of LVSD at 6 months with an increasing duration of the QRS complex on admission (OR for the upper vs. combined lower and middle terciles 1.59; 95% CI 0.80–3.17; *p* = 0.180) and after PCI (OR for the upper vs. combined lower and middle terciles 3.42; 95% CI 1.76–6.66; *p* < 0.001). We also noticed significantly lower values of LVEF in the highest tercile of baseline and post-PCI QRS duration, compared with the lowest and middle terciles (see Figure 1).

### 3.7. Predictors of the Presence of LVSD 6 Months after Discharge from Hospital

**Figure 2:**
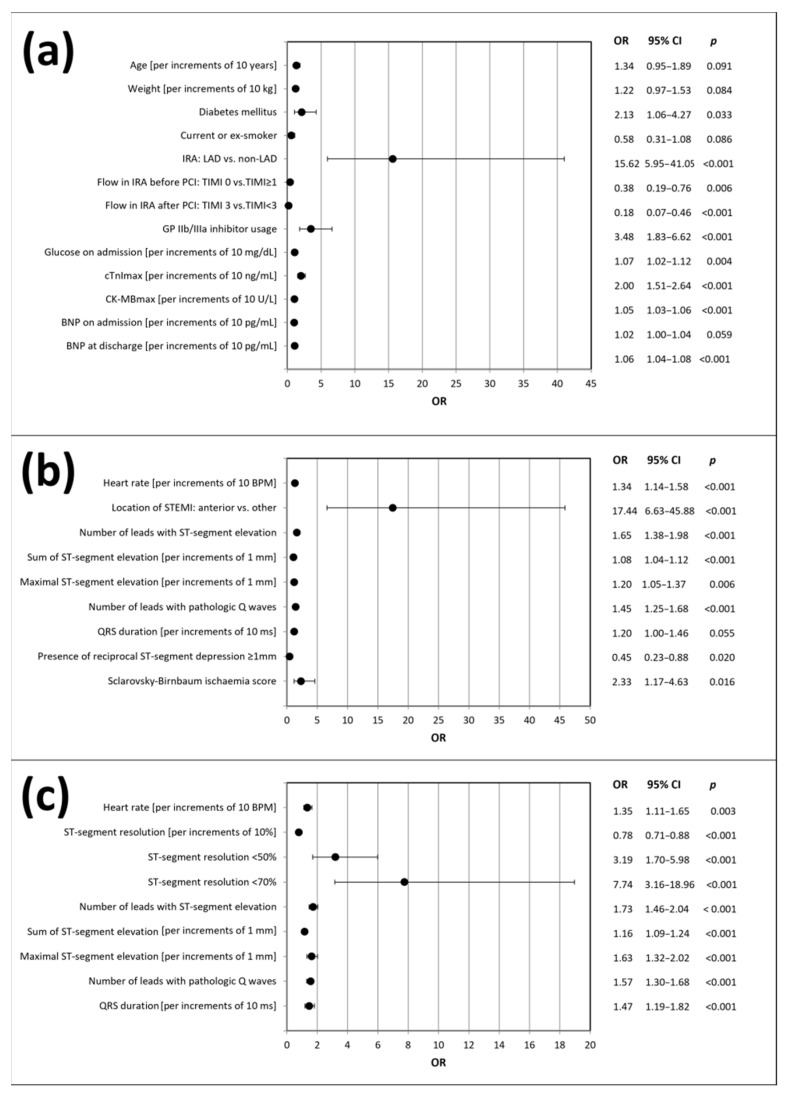
Predictors of LVSD occurrence after 6 months of follow-up according to the univariate logistic regression analysis: (**a**) demographic, clinical, angiographic and biochemical variables; (**b**) baseline electrocardiographic variables; (**c**) post-PCI electrocardiographic variables. BNP, B-type natriuretic peptide; CK-MBmax, maximal activity of isoenzyme MB of creatinine kinase; cTnImax, maximal concentration of troponin I; CI, confidence interval; IRA, infarct-related artery; LAD, left anterior descending artery; LVSD, left ventricular systolic dysfunction; ms, milliseconds; OR, odds ratio; PCI, percutaneous coronary intervention; STEMI, ST-segment elevation myocardial infarction; TIMI, thrombolysis in myocardial infarction score.

Initially, we performed a univariate regression analysis, including electrocardiographic parameters and the variables from Table 1, to identify possible predictors of LVSD after 6 months. Unadjusted models are summarized in Figure 2. Our results indicate strong association of LVSD after 6 months with the majority of electrocardiographic parameters assessed at the time of presentation to hospital and post PCI. Only baseline QRS duration did not show statistical significance. Of note, reciprocal ST-segment depression ≥ 1mm at baseline pointed to a lower likelihood of LVSD after 6 months.

Among the analyzed angiographic variables, LAD as the IRA and usage of GP IIb/IIIa inhibitor were identified as predictors of LVSD occurrence, while TIMI 0 flow before PCI and TIMI 3 flow post PCI were associated with a lower incidence of LVSD after 6 months. The biochemical variables predicting LVSD occurrence included glucose concentration on admission, maximal cardiac troponin I concentration and CK-MB activity, as well as BNP concentration at discharge from hospital. The only clinical variable predictive of LVSD was the presence of diabetes mellitus.

**Figure 3:**
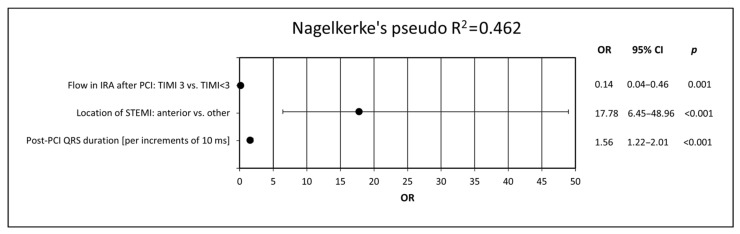
Predictors of LVSD presence after 6 months of follow-up. The model was created using multivariate logistic regression analysis by adding all electrocardiographic variables to the demographic, clinical, angiographic and biochemical data. CI, confidence interval; IRA, infarct-related artery; LVSD, left ventricular systolic dysfunction; ms, milliseconds; OR, odds ratio; PCI, percutaneous coronary intervention; STEMI, ST-segment elevation myocardial infarction, TIMI, thrombolysis in myocardial infarction score.

Next, in order to determine possible independent predictors of LVSD after 6 months, a multivariate logistic regression analysis was performed. We identified anterior location of STEMI, longer post-PCI QRS duration and impaired post-PCI flow in the IRA as the independent predictors of LVSD 6 months after STEMI (Figure 3).

### 3.8. Determinants of LVEF Deterioration

In an attempt to more thoroughly explore the relationship between ECG parameters and LVEF, multiple linear regression analysis with a backward elimination was applied (Table 5). We found that anterior location of STEMI, longer post-PCI QRS duration, higher baseline number of pathological Q-waves and higher baseline Sclarovsky-Birnbaum ischemia score, together with impaired post-PCI flow in the IRA, higher values of body mass index and glucose concentration on admission, were independently associated with lower values of LVEF at 6 months.

## 4. Discussion

### 4.1. General Findings and Study Strengths

According to our results, the majority of the analyzed electrocardiographic parameters measured at baseline and directly after PCI were associated with LVSD 6 months after STEMI. However, when we considered demographic, clinical, angiographic and biochemical characteristics of our study participants, among all assessed ECG parameters, only anterior location of STEMI and longer post-PCI QRS duration remained independent predictors of post-MI LVSD. Additionally, in our study, anterior location of STEMI, longer post-PCI QRS duration, higher baseline number of pathological Q-waves and higher baseline Sclarovsky-Birnbaum ischemia score, together with impaired post-PCI flow in the IRA, higher values of body mass index and glucose concentration on admission, were independently associated with lower LVEF at 6 months.

All electrocardiographic parameters selected for our analysis have been described in the literature to have some predictive value towards LVEF and LVSD. However, many of these reports come from the thrombolysis era and from inhomogeneous cohorts of patients. Our study cohort is characterized by homogeneity concerning the form of acute coronary syndrome presentation (exclusively patients with a first STEMI), reperfusion therapy (exclusively primary PCI) and subsequent pharmacotherapy [18]. This uniformity in study cohort profile, in conjunction with appropriate inclusion and exclusion criteria, allowed us to avoid many potential confounders and enables the extrapolation of these results to the majority of contemporary patients with a first STEMI. Importantly, besides multiple ECG parameters, we examined the impact of numerous demographic, clinical, angiographic and biochemical variables on LVSD occurrence.

### 4.2. Heart Rate

Increased heart rate is a well-recognized risk factor for all-cause and cardiovascular mortality in the general population [29,30,31,32,33,34,35], as well as in patients with stable coronary disease [36,37,38,39,40], heart failure [41,42,43,44,45] and MI [46,47,48]. In STEMI patients, heart rate > 70 bpm recorded at hospital discharge was associated with 2-fold higher 1 year and 4 year mortality rates, while a 5 bpm increment of heart rate was considered to enhance 1 year and 4 year mortality by 29% and 24%, respectively [49]. As reflected by a U-shaped curve, extreme heart rate values (both high and low) are associated with increased mortality [50].

However, we have not found any relevant reports in the literature on the relation of heart rate in the early phase of STEMI with LVEF and the development of post-MI LVSD.

### 4.3. STEMI Location

Anterior wall location of STEMI is a strong independent predictor of bad prognosis, including death [51] and the occurrence of cardiogenic shock in the course of STEMI [52,53], even in the era of primary PCI for STEMI. Associations between anterior location of STEMI and more common development of LVSD [10,11] and left ventricular remodeling [54] have also been reported; however, they were not seen in all studies [55].

### 4.4. ST-Segment-Elevation-Related Parameters

We also evaluated three parameters related to ST-segment elevation (number of leads with ST-segment elevation, sum of ST-segment elevations in all leads, maximal ST-segment elevation in a single lead). According to Rodríguez-Palomares et al., the first two of the three parameters measured in pre-PCI ECG correlated with the size of myocardium at risk [56]. In research by Manes et al., the sum of ST-segment elevation, maximal ST-segment elevation and the number of leads with ST-segment elevation ≥1 mm in predischarge ECG in patients with anterior STEMI predicted a lower probability of recovery of left ventricular function after 90 days [17]. The 3 ST-segment-related parameters have been shown to predict post-STEMI mortality [50,57]. The amplitude of ST-segment elevation was found to be an independent predictor of post-MI 30-day mortality, particularly for total amplitudes ≥ 15 mm [58], and a marker of coronary microcirculation obstruction, performing even superior to ST-segment resolution [59]. It also predicted lack of improvement in left ventricular systolic function after STEMI in 6-month follow-up [60].

### 4.5. ST-Segment Resolution

Resolution of ST-segment elevation of ≥50% is considered a reliable indicator of patency of the IRA. However, restoration of myocardial tissue perfusion occurs only when complete (≥70%) ST-elevation resolution is achieved. Complete (≥70%) resolution of ST-segment elevation predicts lower 1–3 year mortality and lower rates of cardiovascular adverse events [61,62,63] and was associated with better preservation of left ventricular function in comparison with partial (30–70%) or no (<30%) ST-segment resolution. The beneficial outcome of early complete resolution of ST-segment elevation can be seen even after successful primary PCI, with early (i.e., directly after PCI) assessment being more precise in terms of predicting cardiovascular adverse events than assessment after 90 min [64,65,66,67]. Additionally, patients with such early ST-segment resolution also had higher LVEF in comparison to those who achieved ST-segment resolution after 90 min [64]. Failure to achieve complete ST-segment resolution also determined higher peak creatine kinase levels and more common prevalence of significant LVSD [68].

### 4.6. Reciprocal ST-Segment Depression

Besides ECG changes recorded in leads overlying the area of STEMI, the presence of reciprocal ST-segment depressions at baseline may also hold prognostic value. In literature reports it reflected larger infarct area and multivessel coronary artery disease and was associated with increased mortality and higher rates of heart failure, cardiogenic shock and second- and third-degree heart block in a manner proportional to their extent and amplitude [69,70]. Additionally, sustained ST-segment depressions after PCI are predictive of increased mortality after STEMI [71]. We have found no literature reports concerning associations of reciprocal ST-segment depressions with LVSD.

### 4.7. Pathological Q-Waves

The number of leads with pathological Q-waves is another well-recognized predictor of post-MI mortality. It successfully predicted lack of recovery of left ventricular systolic function (defined as absolute LVEF improvement by <10%) within 6 months after STEMI [60]. The presence of pathological Q-waves on the admission ECG is predictive of increased mortality, heart failure and cardiogenic shock after STEMI [3,4,72]. According to Lopez-Castillo et al., the sum of Q-wave depth at discharge performs better than the number of leads with pathological Q-waves as an independent predictor of LVSD development [73].

### 4.8. Sclarovsky-Birnbaum Ischemia Score

Based on the morphology of the terminal portion of the QRS complex and the relative magnitude of ST-segment elevation, the score identifies three grades of ischemia, with grade 3 reflecting most severe ischemia [74] and being an independent predictor of no-reflow phenomenon [67] and mortality [75,76]. Compared with grade 2, it indicates a more extensive infarction area and a higher rate of mortality, heart failure and reinfarction [67,76,77,78,79,80,81,82,83,84]. In terms of left ventricular systolic function, grade 3 of ischemia was associated with lower LVEF [82,85] and a higher incidence of LVSD [67].

### 4.9. QRS Duration

Prolonged QRS duration is another well-established predictor of increased mortality in STEMI patients [86,87,88]. The detrimental effects can already be seen with QRS duration of ≥100 ms [89]. An increase in 30-day mortality was even found for prolongation of QRS duration still within normal ranges (100 ms vs. 80 ms) [50]. Literature reports documenting the relation between QRS duration and LVSD are much scarcer; however, they point to prolonged QRS duration, and even QRS duration of ≥100 ms, as a predictor of LVSD [16,90].

### 4.10. Detailed Analysis of the Study Results

As one can surmise from the above review of the prognostic value of the electrocardiographic parameters, the majority of the literature concerns their association with mortality, while there is a scarcity of data concerning associations with LVSD or LVEF. This fact precludes direct comparison of our results with the cited investigations. However, recognizing LVSD as a surrogate for cardiovascular mortality, the results of our investigation support the prognostic value of ECG regarding prognosis assessment following STEMI.

The results of our investigation basically support the data from the literature. Our study participants who presented with LVSD 6 months after STEMI, in comparison to those without LVSD, had significantly higher values of heart rate, number of leads with ST-segment elevation and pathological Q-waves, sum of ST-segment elevation and maximal ST-segment elevation on admission to hospital and directly after PCI. They also showed a higher prevalence of anterior STEMI and considerably wider QRS after PCI, while QRS duration measurement at baseline showed no significant difference. Additionally, patients presenting with LVSD after 6 months showed more severe ischemia on admission, as assessed with Sclarovsky-Birnbaum ischemia score, smaller reciprocal ST-segment depression at baseline and less profound ST-segment resolution post PCI. 

In univariate analysis, all but one of the ECG parameters predicted LVSD occurrence 6 months after STEMI, the most powerful being anterior location of STEMI (OR 17.44; 95% CI 6.63–45.88). Our analysis also indicates good predictive value of ST-segment resolution and grade 3 according to the Sclarovsky-Birnbaum ischemia score, which came in as the second and third most powerful LVSD predictors. However, it is important to remember that some of the remaining parameters were reported per increments, which means that their actual final impact potentiates when the increments are multiplied in measurements. The only exception was QRS duration at baseline, which did not show statistical significance. In contrast to literature data, in our investigation, the presence of reciprocal ST-segment depressions diminished the likelihood of LVSD occurrence 6 months after STEMI. Whether this could be a consequence of shorter time-to-balloon delay in this group, compared with patients without reciprocal ST-segment depressions, remains a matter of speculation and requires verification in a larger group since the difference in time-to-balloon between patients with and without LVSD was not statistically significant.

In the model adjusted for demographic, clinical, biochemical and angiographic variables, however, the majority of the ECG parameters did not maintain statistical significance. The only two parameters contributing to the multivariate regression model and thus recognized as independent predictors of LVSD in 6-month follow-up were anterior location of STEMI (OR 17.78; 95% CI 6.45–48.96; *p* < 0.001) and post-PCI QRS duration (OR 1.56; 95% CI 1.22–2.00; *p* < 0.001) expressed per increment of 10 ms. The highest tercile of post-PCI QRS duration (i.e., ≥100 ms) was associated with the highest prevalence of LVSD after 6 months and therefore appears to have the best discriminative value. The highest terciles of baseline and post-PCI QRS duration were also associated with significantly lower values of LVEF, compared with lower terciles.

### 4.11. Study Limitations

There are some limitations of this study to be mentioned. First, the study population is a fraction of the original cohort of patients recruited between 2005 and 2008. The time that had elapsed from patient recruitment to the onset of the project and clinical practice modifications implemented over that time could possibly impact the results. Second, LVSD, used as the endpoint in our investigation, is a well-documented prognostic factor in post-STEMI patients; however, it is still a surrogate of clinical endpoints. The choice of LVSD as an endpoint was dictated by lack of power of this study to evaluate clinical endpoints. Third, the duration of follow-up in our research was restricted to 6 months. It seems likely that extending this period might render more favorable results in terms of predictive capabilities of ECG. Forth, the relatively moderate left ventricular systolic function impairment and the applied exclusion criteria noticeably blunting the risk of death and the rate of adverse cardiovascular outcomes in our study group, together with the relatively short time to reperfusion, limit the applicability of our results to all STEMI patients. This warrants further research in non-uniform STEMI cohorts before unrestricted extrapolation of our findings to the general population is feasible. Fifth, enhanced precision of evaluation of left ventricular systolic function, size of myocardial necrosis and patency of the coronary microcirculation could possibly be achieved by employing magnetic resonance imaging. Sixth, we routinely used neither fractional flow reserve measurement nor intravascular ultrasound for the assessment of non-culprit lesions. Seventh, non-critical, non-culprit coronary lesions (stenoses 70–90%) were revascularized electively (within 1 month of the index hospital admission). This fact might have some impact on the study findings. Eighth, in a substantial number of our study participants, maximal concentration of cardiac troponin I exceeded 50 ng/mL. These serum samples were not further diluted, preventing precise estimation of the biochemical infarct size. Ninth, patients with left bundle branch block, isolated posterior myocardial infarction, isolated right ventricular myocardial infarction or permanent atrial fibrillation were excluded from the study. Therefore, the study findings may not to be attributable to such patients. Finally, post-reperfusion ECG parameters in time points other than directly after PCI were not assessed.

## 5. Conclusions

According to our study, baseline and post-PCI ECG parameters possess a modest predictive value for LVSD occurrence within 6 months of a first STEMI.

## Figures and Tables

**Table 1:** Clinical characteristics of the study population in relation to the occurrence of LVSD. Data are presented as median (lower quartile-upper quartile) or number (percent) when appropriate.

Variable	Overall Study Population (*n* = 249)	Patients with LVSD at 6 Months (*n* = 52)	Patients without LVSD at 6 Months (*n* = 197)	*p* *
Age [years]	57.0 (51.0–64.0)	61.0 (52.0–67.0)	56.0 (51.0–64.0)	0.090
Gender [male/female]	186 (74.7%)/63 (25.3%)	42 (80.8%)/10 (19.3%)	144 (73.1%)/53 (26.9%)	0.258
Time from symptom onset to PCI [min]	220.0 (150.0–331.5)	223.5 (148.5–346.0)	220.0 (150.0–321.5)	0.727
**Risk factors for coronary artery disease**				
BMI [kg/m^2^]	26.8 (24.2–29.4)	27.4 (25.0–30.2)	26.5 (24.1–29.1)	0.058
Hypertension	103 (41.4%)	24 (46.2%)	79 (40.1%)	0.431
Diabetes mellitus	50 (20.1%)	16 (30.8%)	34 (17.3%)	0.031
Current or ex-smoker	164 (65.9%)	29 (55.8%)	135 (68.5%)	0.084
Positive family history of IHD	61 (24.5%)	10 (19.2%)	51 (25.9%)	0.321
**Angiographic characteristics**				
IRA: LAD/other	121 (48.6%)/128 (52.4)	47 (90.4%)/5 (9.6%)	74 (37.6%)/123 (62.4%)	<0.001
IRA TIMI 0 flow prior to PCI	144 (57.8%)	39 (75.0%)	105 (53.3%)	0.005
IRA TIMI 3 flow post PCI	229 (92.0%)	41 (78.8%)	188 (95.4%)	0.001
Multivessel coronary artery disease	143 (57,4%)	34 (65.4%)	109 (55.3%)	0.192
Stent implantation	245 (98.4%)	51 (98.1%)	194 (98.5%)	0.678
GP IIb/IIIa inhibitor usage	66 (26.5%)	25 (48.1%)	41 (21.0%)	<0.001
**Biochemical characteristics**				
eGFR (CKD-EPI equation) [mL/min/1.73 m^2^]	84.4 (74.1–94.5)	80.3 (72.8–88.1)	86.5 (75.0–96.6)	0.036
Glucose on admission [mg/dL]	138.5 (122.0–169.0)	157.0 (133.0–193.0)	135 (118.0–168.0)	0.001
cTnI_max_ [ng/mL]	41.2 (11.8–50.0)	50.0 (50.0–50.0)	29.1 (9.7–50.0)	<0.001
CK-MB_max_ [U/L]	242.0 (116.5–414.0)	489.0 (361.5–747.0)	178.5 (95.0–347.5)	<0.001
Total cholesterol [mg/dL]	223.0 (195.0–251.0)	223.0 (195.0–252.0)	223.0 (195.0–251.0)	0.688
LDL-C [mg/dL]	145.0 (125.0–173.0)	145.0 (131.5–170.0)	146.0 (124.0–174.0)	0.712
HDL-C [mg/dL]	52.0 (46.0–59.0)	51.0 (43.0–56.0)	52.0(46.0–59.0)	0.128
Triglycerides [mg/dL]	82.0 (59.0–128.0)	89.5 (62.5–130.5)	78.0(58.0–125.0)	0.103
BNP on admission [pg/mL]	53.9 (27.9–106.5)	74.8 (31.8–155.7)	50.6(27.3–101.9)	0.045
BNP at discharge [pg/mL]	139.8 (74.7–284.2)	436.7 (223.6–735.5)	111.9 (65.3–198.3)	<0.001

BMI, body mass index; BNP, B-type natriuretic peptide; CKD-EPI, Chronic Kidney Disease Epidemiology Collaboration; CK-MB_max_, maximal activity of isoenzyme MB of creatinine kinase; cTnI_max_, maximal activity of troponin I; eGFR, estimated glomerular filtration rate; HDL-C, high-density-lipoprotein cholesterol; IHD, ischemic heart disease; IRA, infarct-related artery; LAD, left anterior descending artery; LDL-C, low-density-lipoprotein cholesterol; LVSD, left ventricular systolic dysfunction; PCI, percutaneous coronary intervention; TIMI, thrombolysis in myocardial infarction score. * for comparison between groups with and without LVSD at 6 months.

**Table 2:** Echocardiographic characteristics of the study population in relation to LVSD occurrence. Data are presented as median (lower quartile-upper quartile).

Variable	Overall Study Population (*n* = 249)	Patients with LVSD at 6 Months (*n* = 52)	Patients without LVSD at 6 Months (*n* = 197)	*p* *
**At discharge**				
LA [mm]	40.0 (37.0–43.0)	41.0 (38.0–45.0)	39.0 (37.0–42.0)	0.007
LVEDd [mm]	49.0 (45.0–53.0)	53.0 (49.0–56.0)	47.0 (45.0–52.0)	<0.001
LVESd [mm]	34.0 (30.0–37.0)	38.0 (35.0–40.5)	33.0 (30.0–36.0)	<0.001
LVEDV [mL]	99.4 (84.0–121.0)	121.5 (102.5–132.5)	93.0 (81.0–111.0)	<0.001
LVESV [mL]	55.0 (45.0–69.0)	75.0 (66.0–84.5)	51.0 (42.5–62.0)	<0.001
LVEF [%]	44.0 (39.0–48.4)	36.0 (33.5–38.5)	45.9 (42.0–50.0)	<0.001
LVSD (LVEF ≤ 40%)	84.0 (33.7%)	45 (86.5%)	39 (19.8%)	<0.001
WMSI [points]	1.56 (1.38–1.75)	1.88 (1.78–1.94)	1.44 (1.38–1.69)	<0.001
**6 months after discharge**				
LA [mm]	40.0 (38.0–44.0)	44.0 (40.0–46.0)	40.0 (37.0–42.0)	<0.001
LVEDd [mm]	50.0 (46.0–54.0)	55.0 (52.0–57.0)	48.0 (45.0–53.0)	<0.001
LVESd [mm]	34.0 (31.0–37.0)	40.0 (36.0–44.0)	33.0 (31.0–36.0)	<0.001
LVEDV [mL]	110.0 (94.0–134.0)	145.0 (129.5–163.0)	105.0 (91.0–125.0)	<0.001
LVESV [mL]	57.0 (48.0–76.0)	92.0 (79.0–103.0)	53.0 (45.0–65.0)	<0.001
LVEF [%]	46.0 (42.0–51.5)	36.0 (33.7–38.5)	48.0 (44.8–52.5)	<0.001
WMSI [points]	1.44 (1.31–1.69)	1.88 (1.75–1.94)	1.38 (1.31–1.50)	<0.001

LA, left atrium end-systolic diameter; LVEDd, left ventricular end-diastolic diameter; LVEDV, left ventricular end-diastolic volume; LVEF, left ventricular ejection fraction; LVESd, left ventricular end-systolic diameter; LVESV, left ventricular end-systolic volume; LVSD, left ventricular systolic dysfunction; WMSI, wall motion score index. * for comparison between groups with and without LVSD at 6 months.

**Table 3:** Occurrence of LVEF ≤ 40 % on transthoracic echocardiography at hospital discharge and at 6 months.

		LVEF ≤ 40 % (LVSD) at 6 Months	
		Absent (*n* = 197)	Present (*n* = 52)
**LVEF ≤40 %** **at hospital discharge**	Absent (*n* = 165)	158 (63.5%)	7 (2.8%)
	Present (*n* = 84)	39 (15.7%)	45 (18.1%)

LVEF, left ventricular ejection fraction; LVSD, left ventricular systolic dysfunction.

**Table 4:** Electrocardiographic characteristics of the study population in relation to LVSD occurrence. Data are presented as median (lower quartile-upper quartile) or number (percent) when appropriate.

Variable	Overall Study Population (*n* = 249)	Patients with LVSD at 6 Months (*n* = 52)	Patients without LVSD at 6 Months (*n* = 197)	*p* *
**Baseline**				
Heart rate [BPM]	75.0 (62.0–88.0)	81.0 (68.5–97.0)	74.0 (60.0–85.0)	<0.001
Anterior location of STEMI	116 (47.0%)	47 (90.4%)	69 (35.0%)	<0.001
Number of leads with ST-segment elevation [*n*]	4.0 (3.0–6.0)	6.0 (5.0–7.0)	3.0 (3.0–5.0)	<0.001
Sum of ST-segment elevation [mm]	8.5 (4.0–14.0)	13.8 (9.8–18.0)	7.0 (4.0–12.0)	<0.001
Maximal ST-segment elevation [mm]	3.0 (2.0–4.0)	3.5 (3.0–5.0)	2.5 (1.5–4.0)	<0.001
Number of leads with pathologic Q waves [*n*]	2.0 (1.0–4.0)	4.0 (3.0–5.0)	2.0 (1.0–3.0)	<0.001
Presence of reciprocal ST-segment depression ≥ 1mm	193 (77.5%)	34 (65.4%)	159 (80.7%)	0.019
QRS duration [ms]	95.0 (85.0–100.0)	95.0 (86.0–110.0)	95.0 (85.0–100.0)	0.399
Sclarovsky-Birnbaum ischemia score	grade 2: 198 (79.5%); grade 3: 51 (20.5%)	grade 2: 35 (67.3%) grade 3: 17 (32.7%)	grade 2: 163 (82.7%); grade 3: 34 (17.3%)	0.014
**Post PCI**				
Heart rate [BPM]	77.0 (66.0–89.0)	83.0 (72.0–94.0)	75.0 (64.0–88.0)	0.003
ST-segment resolution [%]	60.6 (30.0–88.9)	39.4 (0.0–69.3)	70.0 (40.0–100.0)	<0.001
ST-segment resolution (≥50%)	160 (64.3%)	22 (42.3%)	138 (70.1%)	<0.001
ST-segment resolution after PCI (trichotomised)	<30%–62 (24.9%) ≥30–69%–82 (32.9%) ≥70%–105 (42.2%)	<30%–22 (42.3%) ≥30–69%–24 (46.2%) ≥70%–6 (11.5%)	<30%–40 (20.3%) ≥30–69%–58 (29.4%) ≥70%–99 (50.3%)	<0.001
Number of leads with ST-segment elevation [*n*]	3.0 (1.0–5.0)	5.5 (4.0–7.0)	3.0 (0.0–4.0)	<0.001
Sum of ST-segment elevation [mm]	3.0 (1.0–7.0)	8.3 (5.0–13.0)	2.0 (0.0–4.5)	<0.001
Maximal ST-segment elevation [mm]	1.0 (0.5–2.0)	2.3 (1.5–4.0)	1.0 (0.5–1.5)	<0.001
Number of leads with pathologic Q waves [*n*]	3.0 (2.0–5.0)	5.0 (4.0–7.0)	3.0 (1.0–4.0)	<0.001
QRS duration [ms]	90.0 (84.0–100.0)	99.5 (87.5–111.0)	90.0 (83.0–100.0)	0.003

BPM, beats per minute; LVSD, left ventricular systolic dysfunction; PCI, percutaneous coronary intervention; STEMI, ST-segment elevation myocardial infarction. * for comparison between groups with and without LVSD at 6 months.

**Table 5:** Impact of demographic, clinical, angiographic, biochemical and electrocardiographic variables on left ventricular ejection fraction (LVEF) 6 months after STEMI. The model was obtained using multiple regression by adding all electrocardiographic variables to the demographic, clinical and angiographic data.

Variable	Beta Coefficient	Beta Coefficient Standard Error	Direction Component Beta	Direction Component Beta Standard Error	*p*
Model characteristics: R = 0.682; R^2^ = 0.464; corrected R^2^ = 0.448; *p* < 0.00001					
Intercept			70.49	3.71	<0.0001
BMI [kg/m^2^]	−0.10	0.05	−0.20	0.10	0.0461
Glucose on admission [per increments of 10 mg/dL]	−0.16	0.05	−0.21	0.07	0.0021
IRA TIMI 3 flow after PCI	0.12	0.05	−3.20	1.36	0.0196
Anterior location of STEMI	−0.36	0.05	−5.45	0.83	<0.0001
QRS duration on admission [per increments of 10 ms]	−0.28	0.05	−1.36	0.25	<0.0001
Number of leads with pathologic Q waves on admission	−0.24	0.05	−0.82	0.18	<0.0001
Sclarovsky-Birnbaum ischemia score [grade 3 vs. grade 2]	−0.12	0.05	−2.33	0.94	0.0137

BMI, body mass index; IRA, infarct-related artery; PCI, percutaneous coronary intervention; STEMI, ST-segment elevation myocardial infarction; TIMI, thrombolysis in myocardial infarction score.

## Data Availability

Data sharing is not applicable to this article.
